# The Loop-In Binding Mode of Dihydroorotase: Implications for Ligand Binding and Therapeutic Targeting

**DOI:** 10.3390/ijms26031359

**Published:** 2025-02-06

**Authors:** Cheng-Yang Huang

**Affiliations:** 1 Department of Biomedical Sciences, Chung Shan Medical University, Taichung City 402, Taiwan; cyhuang@csmu.edu.tw; 2 Department of Medical Research, Chung Shan Medical University Hospital, Taichung City 402, Taiwan

**Keywords:** dihydroorotase, CAD, anticancer, loop, 5-fluoroorotate, plumbagin, 5-aminouracil, 5-fluorouracil

## Abstract

Dihydroorotase (DHOase; EC 3.5.2.3) is a zinc-dependent metalloenzyme that plays a key role in the de novo pyrimidine biosynthesis pathway, catalyzing the reversible cyclization of *N*-carbamoyl aspartate to dihydroorotate. This reaction is essential for the production of uridine monophosphate, the precursor of all pyrimidine nucleotides required for DNA and RNA synthesis. Despite its conserved enzymatic function, DHOase exhibits significant structural diversity across species, particularly in its oligomeric states, gene fusion patterns, and active site architecture. A crucial structural feature of DHOase is its flexible active site loop, which undergoes dynamic conformational changes during catalysis. Previously, the loop-in conformation was associated with substrate binding, whereas the loop-out conformation was linked to product release and non-substrate ligand binding. However, recent crystallographic studies challenge this paradigm, revealing that certain non-substrate ligands and inhibitors, including malate, 5-fluoroorotate, plumbagin, 5-aminouracil, and 5-fluorouracil, interact with DHOase via a loop-in binding mechanism rather than the previously assumed loop-out mode. These findings necessitate a reassessment of the catalytic mechanism of DHOase and underscore the active site loop as a potential target for drug development. This review revisits the structural and biochemical mechanisms of DHOase, with a focus on recent crystallographic insights that redefine the loop-in binding mode for ligand interaction. By leveraging the unique conformational dynamics of the active site loop, novel inhibitors may be developed to selectively target pyrimidine biosynthesis in cancer cells and microbial pathogens. These insights emphasize the crucial role of structural biology in therapeutic design and highlight DHOase as a promising drug target.

## 1. Introduction

**Figure 1:**
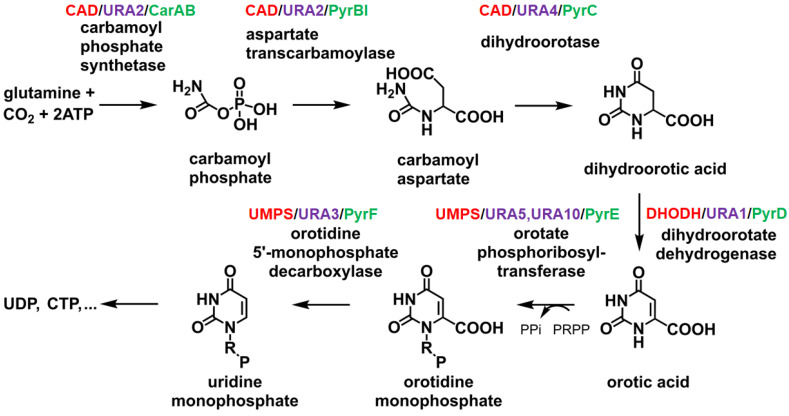
The process of de novo synthesis of pyrimidine nucleotides. UMP serves as the precursor for all pyrimidine nucleotides, including UDP, CTP, and others, with its synthesis involving six enzymatic steps. In animals (highlighted in red), the first three steps are catalyzed by a trifunctional, cytoplasmic enzyme known as CAD. The name “CAD” is derived from the three enzymatic activities present in this protein: carbamoyl phosphate synthetase (CPSase), aspartate transcarbamylase (ATCase), and DHOase. In bacteria (highlighted in green), these three enzymatic activities are carried out by three separate proteins. In contrast, in yeast (highlighted in purple), only the first two enzymes are fused into a single gene product, URA2.

Dihydroorotase (DHOase; EC 3.5.2.3) is a zinc-dependent metalloenzyme that plays a crucial role in the de novo pyrimidine biosynthesis pathway [1,2,3,4], which is indispensable for the survival and proliferation of all living organisms. This pathway provides the essential building blocks required for DNA and RNA synthesis [5,6,7,8,9]. DHOase catalyzes the reversible cyclization of *N*-carbamoyl aspartate to dihydroorotate (Figure 1), representing the third step in a cascade of enzymatic reactions culminating in the synthesis of uridine monophosphate (UMP)—the precursor for all pyrimidine nucleotides [9,10]. Pyrimidine nucleotides derived from UMP are fundamental to a broad spectrum of biological processes. Beyond their critical roles in RNA and DNA synthesis, they are integral to the production of phospholipids and the glycosylation of proteins, both of which are vital for maintaining cell membrane integrity and facilitating cellular signaling [2,11]. Additionally, pyrimidine nucleotides are crucial activators of intermediates in glycogen metabolism, highlighting their versatile biological importance [12,13,14,15]. Given its central role in nucleotide biosynthesis, DHOase is often tightly regulated to maintain cellular homeostasis [6].

Although the de novo synthesis of pyrimidine nucleotides is highly conserved across nearly all organisms, the structural and organizational diversity of the enzymes involved, particularly DHOase, is remarkable. DHOase exhibits substantial variations across species in terms of subunit composition, oligomerization state, and the structural organization of active site loops. These differences offer valuable opportunities for species-specific drug targeting. Of particular interest is the active site loop of DHOase, which plays a key role in substrate binding and catalysis. Its conformational flexibility, especially in the context of ligand interactions, makes it a promising target for drug design. In this review, we aim to provide an overview of the current understanding of DHOase, focusing on the recently identified loop-in binding mode for ligand interaction, as revealed by new structural evidence.

## 2. DHOase Enzymes

### 2.1. Physiological Roles and Disease Implications of DHOase

DHOase plays an integral role in the de novo pyrimidine biosynthesis pathway, which is critical for generating the nucleotides required for cellular proliferation and maintaining metabolic homeostasis [6]. In rapidly dividing cells, such as those in tumors [4,16,17,18,19,20] or parasitic infections [5,21,22,23], this pathway is upregulated to meet the heightened demand for DNA and RNA synthesis. Consequently, DHOase activity becomes a focal point for maintaining the pyrimidine nucleotide pool, which is indispensable for cell growth, survival, and division. In cancer biology, the overexpression of CAD, the multifunctional enzyme complex that includes DHOase, has been linked to poor prognosis in aggressive cancers such as glioblastoma [17,24] and hepatocellular carcinoma [25,26,27,28,29]. The upregulation of CAD facilitates tumor growth by ensuring a continuous supply of pyrimidine nucleotides. The inhibition of this pathway has shown promise in reducing tumor cell proliferation and inducing apoptosis. Moreover, CAD-mediated pyrimidine biosynthesis is not only essential for cancer progression but also plays a role in immune evasion, as some studies suggest a correlation between pyrimidine metabolism and immune checkpoint activity [8,30]. Beyond oncology, DHOase and CAD mutations are implicated in rare genetic disorders such as early infantile epileptic encephalopathy [31,32,33,34,35,36,37,38]. This condition is characterized by seizures, developmental delays, and anemia, attributed to defective pyrimidine biosynthesis. In these patients, uridine supplementation, which bypasses the need for de novo pyrimidine synthesis, has proven effective in ameliorating symptoms, underscoring the critical physiological role of CAD and DHOase. In infectious diseases, DHOase also plays a pivotal role. Pathogens such as *Toxoplasma gondii* [21] and *Plasmodium falciparum* [39,40,41,42,43] rely heavily on the de novo pyrimidine biosynthesis pathway due to their limited ability to salvage pyrimidines from the host. This makes DHOase an attractive drug target for antiparasitic therapies. Inhibiting DHOase has been shown to significantly impair parasite growth and reduce virulence in models of toxoplasmosis and malaria. Given its multifaceted role in cell biology, disease progression, and metabolism, DHOase represents a nexus of therapeutic opportunities. Targeting its activity offers the potential to modulate pyrimidine metabolism selectively in pathological conditions, such as cancer, parasitic infections, and metabolic disorders, while minimizing effects on normal, quiescent cells that rely on salvage pathways.

### 2.2. Diversity and Classification of DHOase

**Figure 2:**
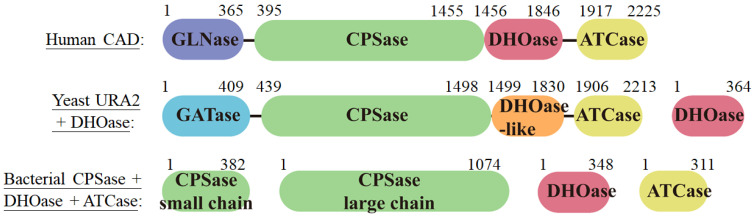
Comparison of DHOases. The gene products involved in the first three reactions of pyrimidine biosynthesis vary among species. In humans, CAD consists of covalently fused domains for DHOase, CPSase, and ATCase activities. In yeast, such as *Saccharomyces cerevisiae*, CPSase and ATCase activities are combined in a single bifunctional protein, URA2. URA2 is a CAD-like polypeptide that includes a defective DHOase-like domain. In bacteria, such as *Escherichia coli*, CPSase, DHOase, and ATCase are expressed as separate proteins that function independently. The labeled numbers indicate the domain lengths in amino acid residues. Despite differences in URA2, yeast DHOase belongs to type II, similar to *E. coli* DHOase.

DHOase activity is found in all organisms and is essential for the biosynthesis of pyrimidine nucleotides. One might expect DHOase to be highly conserved throughout evolution. However, phylogenetic and structural analyses have revealed at least three distinct forms of DHOase [44]. Type I DHOases, which are evolutionarily ancient and larger (approximately 45 kDa), include those from Gram-positive bacteria such as *Staphylococcus aureus* (SaDHOase) [45], *Bacillus anthracis* (BaDHOase) [46], and *Aquifex aeolicus* (AaDHOase) [47]. In contrast, type II DHOases are smaller (approximately 38 kDa) and are found in most eubacteria (e.g., *Escherichia coli* and *Klebsiella pneumoniae*), fungi, and plants [48,49,50]. Recent structural analyses suggest that human DHOase domain (huDHOase) of CAD protein should be reclassified from type I to type III DHOase [44]. In mammals, DHOase is part of a trifunctional polypeptide, CAD [51,52,53], which includes carbamoyl phosphate synthetase (CPSase) and aspartate transcarbamoylase (ATCase) (Figure 2). This CAD protein has a molecular weight of 240 kDa and self-assembles into a 1.5-MDa hexamer [2]. In yeast, such as *Saccharomyces cerevisiae*, CPSase and ATCase are combined in a bifunctional protein named URA2, a CAD-like polypeptide that contains a defective DHOase-like domain [49,54]. Yeast DHOase, however, remains a monofunctional enzyme and is classified as type II DHOase [55]. In most prokaryotes, CPSase, ATCase, and DHOase are expressed as monofunctional enzymes, functioning independently [11,50]. Despite the near-universal presence of DHOase activity in living systems, it is both surprising and intriguing that the structure, protein length, and gene product fusion of DHOase exhibit such significant diversity across species. These differences raise important questions about the evolutionary pressures and functional adaptations that shaped DHOase variants over time.

### 2.3. Biochemistry and Enzymatic Functions of DHOase

The DHOase reaction represents a critical metabolic checkpoint, linking the earlier condensation of carbamoyl phosphate and aspartate, catalyzed by ATCase, to the downstream oxidation of dihydroorotate by dihydroorotate dehydrogenase (Figure 1) [2]. The activity of bacterial DHOase depends on a conserved active site containing two zinc ions, while huDHOase uniquely contains three zinc ions [44]. These metal ions are coordinated by histidine and aspartate residues, stabilizing reaction intermediates and facilitating the condensation reaction [56,57,58]. This zinc-dependent mechanism ensures high specificity and catalytic efficiency. Mutations affecting the active site residues have been shown to significantly impair enzyme function, disrupting pyrimidine biosynthesis and potentially leading to metabolic disorders [32,59]. In mammals, DHOase is integrated into the CAD protein complex. This integration allows for substrate channeling, minimizing the diffusion of intermediates and enhancing the overall efficiency of pyrimidine nucleotide synthesis [60,61,62,63,64]. Substrate channeling is particularly critical in rapidly dividing cells, where high nucleotide turnover is required for DNA and RNA synthesis. Due to differences in gene product fusion (Figure 2), the interaction modes of DHOase with associated enzymes vary significantly across species [1]. The enzymatic function of DHOase is also subject to regulation by the broader metabolic environment. In microorganisms, its activity may be influenced by interactions with other enzymes in transient complexes [47,65]. In contrast, in mammals, CAD is subject to allosteric modulation and phosphorylation [66]. Phosphorylation of CAD by kinases such as PKA and MAPK modulates its activity in response to cellular signaling, linking pyrimidine biosynthesis to the cell cycle and growth signals. This regulation ensures that pyrimidine production is synchronized with the metabolic and proliferative demands of the cell. Interestingly, DHOase exhibits variability in organization and activity across different species. In many prokaryotes, DHOase functions independently but forms transient complexes with ATCase to enhance catalysis [13]. However, in some organisms, such as *A. aeolicus*, DHOase is only active when in a stable complex with its associated enzyme partners, illustrating how evolutionary adaptations optimize enzymatic activity [47,61,62]. The enzyme’s central role in nucleotide biosynthesis and its intricate regulation highlight its importance in maintaining cellular homeostasis and its potential as a target for therapeutic intervention. Investigating the structural and biochemical properties of DHOase across species is critical to understanding how these enzymes differ. Thus, it is possible to develop species-specific inhibitors that selectively target pathogens or cancer cells without affecting human cells, by exploiting differences in the biochemistry of DHOase between species.

### 2.4. Structural Features and Active Site Dynamics of DHOase

**Figure 3:**
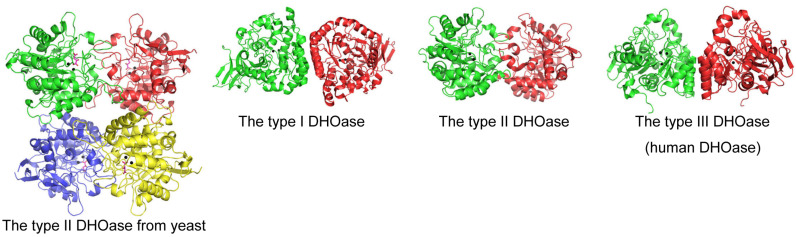
Structures of DHOases. The global architecture of each DHOase monomer reveals a TIM barrel structure. Ribbon diagrams of the yeast *Saccharomyces cerevisiae* DHOase tetramer, the type I enzyme *Bacillus anthracis* DHOase dimer, the type II enzyme *Escherichia coli* DHOase dimer, and the human dimeric DHOase domain are shown, with each monomer color-coded accordingly. Zinc ions in the active site are depicted as black spheres. Despite their similar monomeric structures, the oligomeric states and self-assembly types of DHOases differ significantly across species. These differences are likely to influence their interactions with partner proteins during complex formation. Note that human DHOase may exist as either a monomer or a dimer. This figure was created using PyMOL v2.2.0 software.

**Figure 4:**
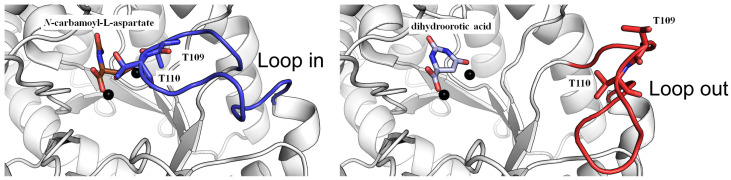
Reaction mechanism of DHOase proposed from the *E. coli* DHOase crystal structure. The crystal structure of dimeric *E. coli* DHOase (PDB ID: 1XGE) reveals two distinct states per monomer within the same dimeric enzyme—one for substrate *N*-carbamoyl-L-aspartate binding (colored in brown) and the other for product dihydroorotic acid release (colored in light blue). Upon substrate binding, the flexible loop (highlighted in blue) closes over the active site (loop-in mechanism), creating a tightly enclosed environment that stabilizes the transition state and enhances catalytic efficiency. Zinc ions in the active site are depicted as black spheres. Following catalysis, the loop reopens (highlighted in red) to facilitate product release (loop-out mechanism). This dynamic “open–close” transition is essential for enzymatic function and represents a potential target for inhibitor design. The loop-in and loop-out binding modes are proposed based on structural analysis of the crystal structure.

The structure of DHOase is characterized by a highly conserved active site that coordinates two or three zinc ions, which are essential for its catalytic activity [1,56]. These zinc ions, together with key active site residues—histidines, an aspartate, and a post-carbamated lysine—form a robust catalytic core that facilitates the cyclization of *N*-carbamoyl aspartate into dihydroorotate. This conserved architecture is further stabilized by a network of hydrogen bonds and hydrophobic interactions, ensuring structural integrity and substrate specificity. Mutations affecting this lysine or the zinc-coordinating residues drastically impair enzymatic activity, highlighting their critical functional significance [67]. Despite similar monomeric structures, the oligomeric states and self-assembly types of DHOase differ significantly across species (Figure 3). The structural organization of DHOase reflects evolutionary adaptations, resulting in distinct interaction mechanisms with partner proteins that remain largely unexplored [59,68]. Another hallmark feature of DHOase is its flexible active site loop [57], which undergoes significant conformational changes during the catalytic cycle (commonly referred to as the loop-in or loop-out binding mechanism) [58]. This loop plays a critical role in substrate recognition, binding, and positioning [69]. Upon substrate binding, the loop closes over the active site (loop-in mechanism), creating a tightly enclosed environment that stabilizes the transition state and enhances catalytic efficiency (Figure 4). Following the reaction, the loop reopens to release the product, dihydroorotate (loop-out mechanism). This dynamic “open–close” mechanism is integral to the enzyme’s function and represents a potential target for inhibitor design. However, recent crystallographic studies have challenged this proposed mechanism [68,70,71,72,73,74]. These findings indicate that a refined or alternative mechanism may exist, requiring further experimental evidence through structural and biochemical analyses. Additionally, the identification of more inhibitors is necessary to build a foundation for future drug development [30,45,73,75]. Comparative structural studies have revealed subtle differences in the active site architecture of DHOase across organisms [1,11,48]. For instance, bacterial DHOase has a more compact active site, potentially allowing for tighter substrate binding. In contrast, mammalian DHOase has evolved features that facilitate integration within the CAD complex and regulation through phosphorylation. These differences provide unique opportunities for the development of species-specific inhibitors that target pathogens or parasitic organisms without affecting host cells. Beyond the catalytic core, DHOase also contains additional structural motifs that contribute to its stability and function. In prokaryotic systems, these motifs mediate protein–protein interactions, enabling the formation of transient enzyme complexes. In mammals, regulatory domains within the CAD complex influence DHOase’s structural conformation, linking its activity to cellular signals such as growth factors and nutrient availability. Phosphorylation of these regulatory regions modulates active site accessibility and enzymatic activity, further illustrating the intricate interplay between structure and function [13,76]. Overall, the phosphorylation, the self-assembly state, and the dynamic nature of the active site loop and its interactions with substrates and regulatory molecules make DHOase a compelling target for drug discovery. Inhibitors that stabilize the loop in an inactive conformation or disrupt zinc ion coordination are highly desirable and warrant further investigation. Due to the limited availability of complexed structures, additional structural evidence is crucial for the rational design of novel therapeutics targeting pyrimidine biosynthesis in cancer and infectious diseases.

### 2.5. DHOase Belongs to the Cyclic Amidohydrolase Family with a Similar Active Site for Catalysis

**Figure 5:**
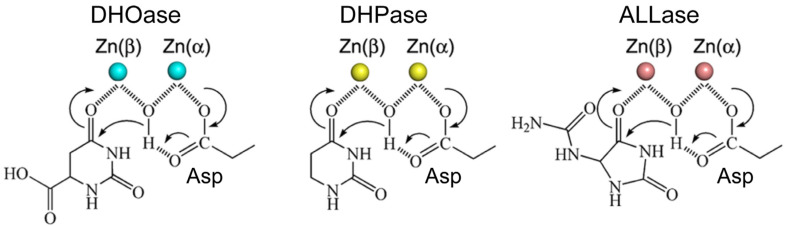
Catalytic mechanism of the cyclic amidohydrolase family. The chemical mechanisms of DHOase, DHPase, and ALLase are similar. However, despite having a similar active site, DHOase cannot utilize the substrates of other cyclic amidohydrolases. The hydrolysis of substrates likely proceeds through three steps: (1) the hydrolytic water molecule is activated by an aspartate residue to perform a nucleophilic attack; (2) the amide bond of the substrate is made more electrophilic through polarization of the carbonyl oxygen bond; and (3) the leaving-group nitrogen is protonated as the carbon–nitrogen bond is cleaved. Metal ions, which are critical for the reaction, are depicted as circles. Note that the reaction catalyzed by DHOase, as described here, corresponds to the reverse reaction when performed at pH 7.0 or higher.

Based on amino acid sequence analysis, DHOase has been identified as a member of the cyclic amidohydrolase family [56,77,78], which also includes dihydropyrimidinase (DHPase) [79,80,81,82,83,84] and allantoinase (ALLase) [85,86,87,88]. These metal-dependent enzymes catalyze the hydrolysis of cyclic amide bonds in substrates with 5- or 6-membered rings, playing a key role in the metabolism of purines and pyrimidines (Figure 5). Most enzymes in this family share a binuclear metal center, coordinated by four histidine residues, one aspartate residue, and a post-translationally carbamylated lysine residue. Although these cyclic amidohydrolases use a similar active site and catalytic mechanism, no substrate overlap has been observed among DHOase, DHPase, and ALLase [75]. The carbamylated lysine is essential for enzyme activity and the assembly of the binuclear metal center [89,90]. However, crystal structural analyses have revealed that in huDHOase, the carbamylated lysine primarily influences the binding of one metal ion to the active site [91]. In addition to variations in the role of the carbamylated lysine, differences in metal dependence have also been observed among cyclic amidohydrolases [56]. For example, *Tetraodon nigroviridis* DHPase is catalytically active with only one zinc ion in its active site [83]. Similarly, *Aquifex aeolicus* DHOase contains only a single zinc ion [92]. In contrast, huDHOase possesses a novel third zinc ion, coordinated by a histidinate ion at the active site, which is functionally important but absent in other DHOases [44]. These findings highlight the need for further structural analyses to clarify the roles of metal ions and the carbamylated lysine residue, as well as to better understand the architecture and functional diversity of different DHOases [93]. Such studies will provide valuable insights into the mechanistic and evolutionary adaptations within this enzyme family.

### 2.6. DHOase as a Drug Target

**Figure 6:**
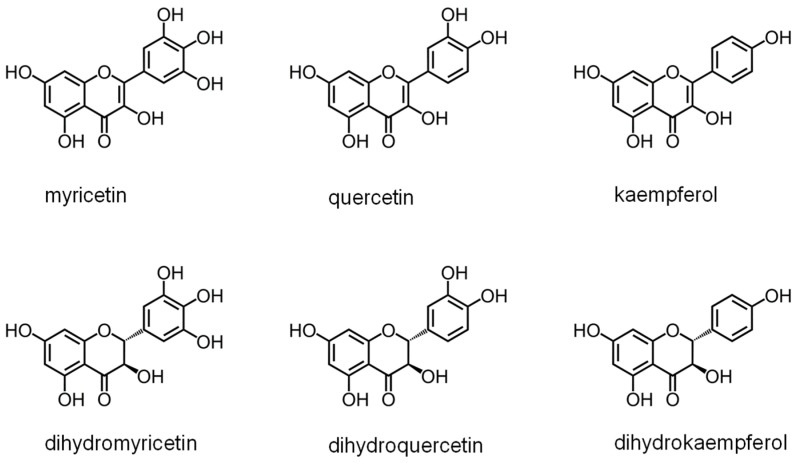
**Structure of some flavonoids.** Kaempferol can inhibit *Klebsiella pneumoniae* DHOase. The inhibitory efficacy of flavonols followed the following order: kaempferol > myricetin > quercetin [75].

**Figure 7:**
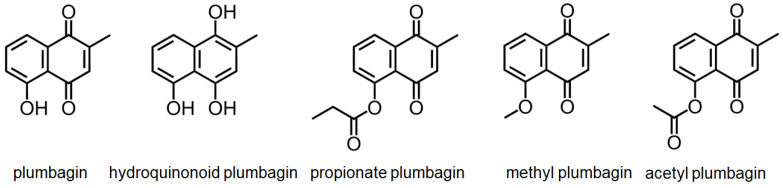
**Structure of plumbagin derivatives.** Plumbagin can inhibit *Klebsiella pneumoniae* DHOase [73]. Whether the derivative analogs can inhibit DHOase activity remains unexplored.

**Figure 8:**
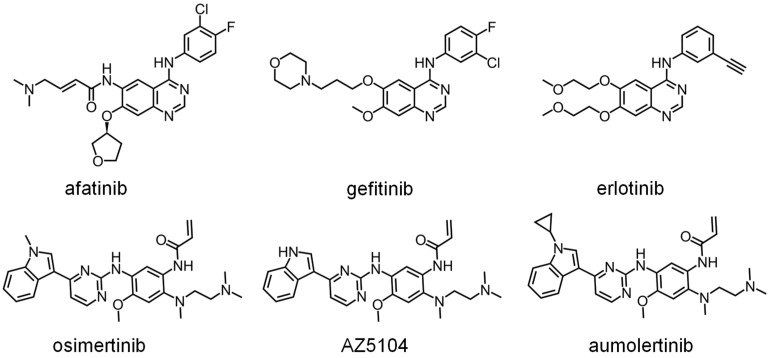
**Structure of some anti-lung cancer drugs.** The lung cancer drug afatinib has been found to inhibit CAD enzyme activity [30]. Whether the derivative analogs and other lung cancer clinical drug can inhibit CAD activity remains unexplored.

**Figure 9:**
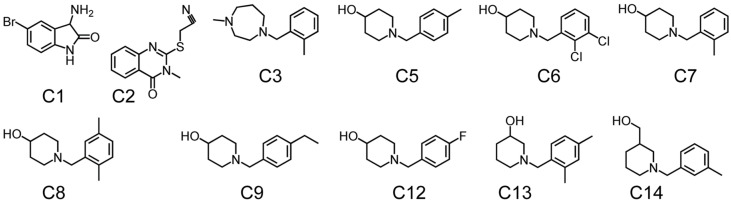
**Structure of 1-benzylpiperidin-4-ol derivatives.** These compounds [46] are synthesized for inhibiting the type I DHOase from *Staphylococcus aureus*.

**Figure 10:**
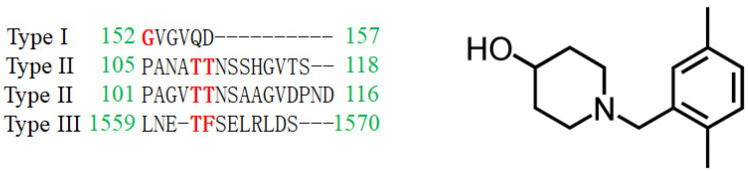
**Sequence alignment of the active site flexible loop and the inhibitor for type I *Staphylococcus aureus* DHOase.** DHOases from *Staphylococcus aureus* (type I), *Escherichia coli* (type II), *Saccharomyces cerevisiae* (type II), and humans (type III) are compared. Amino acids involved in catalysis are highlighted in red. The sequence composition and length of these flexible loops vary significantly. Among them, *Saccharomyces cerevisiae* DHOase has the longest loop. The inhibitor, which contains a 1-benzylpiperidin-4-ol core, is effective against type I DHOases. However, whether it can selectively or universally inhibit all DHOases remains unknown.

DHOase has emerged as a promising target for therapeutic intervention due to its central role in pyrimidine biosynthesis, a pathway critical for the survival and proliferation of rapidly dividing cells. Cancer cells [4,16,17,18,19,94], parasitic organisms [21,41,42], and certain pathogens [5,22,23,45,48] exhibit a heightened dependency on this pathway to meet their increased nucleotide demands, making DHOase an attractive target for selective drug development. In oncology, the upregulation of CAD has been observed in glioblastoma and hepatocellular carcinoma. Pharmacological inhibition of CAD has demonstrated significant anti-proliferative effects by disrupting the nucleotide supply, thereby inducing cell cycle arrest and apoptosis. Since normal, quiescent cells predominantly rely on salvage pathways for nucleotide biosynthesis, targeting DHOase or the CAD complex offers a strategy to selectively inhibit tumor cells while sparing healthy tissues. The active site loop of DHOase, which undergoes conformational changes during catalysis, represents a particularly promising target for drug design [1,68,69,71,72,73,74,75]. Compounds that stabilize this loop in an inactive conformation or disrupt its interaction with the substrate could effectively inhibit enzyme activity. In the context of infectious diseases, pathogens such as *Plasmodium falciparum* and *Toxoplasma gondii* rely heavily on the de novo pyrimidine biosynthesis pathway due to their limited ability to salvage pyrimidines from the host [21,41,42]. Inhibiting DHOase in these organisms has been shown to significantly impair parasite growth and replication, underscoring its potential as a drug target for malaria and toxoplasmosis. Importantly, structural differences in the active site of DHOase between humans and these pathogens provide opportunities for the development of highly selective inhibitors [40,44,45,46,48,73]. DHOase inhibitors are also being explored in antiviral research. Viruses such as hepatitis B [19,95] and SARS-CoV-2 [23] exploit host pyrimidine biosynthesis to support viral genome replication. Targeting host DHOase, either directly or through CAD inhibition, can reduce the availability of nucleotides required for viral replication, offering a host-directed antiviral strategy that is less likely to be undermined by viral mutations. The multifaceted regulation of CAD through phosphorylation, allosteric modulation, and protein–protein interactions further enable the development of drugs that selectively target its activity under pathological conditions [2,28,29,66,76]. Additionally, combining DHOase inhibitors with drugs that block downstream enzymes, such as dihydroorotate dehydrogenase, can create synergistic effects, enhancing therapeutic efficacy in both cancer and infectious disease models [4,24,25,28,39,42]. Recent studies have shown that natural products like kaempferol (Figure 6) and plumbagin (Figure 7) can inhibit *Klebsiella pneumoniae* DHOase [73,75], while the lung cancer drug afatinib (Figure 8) has been found to inhibit CAD enzyme activity [30]. Whether their derivative analogs can inhibit DHOase activity remains unexplored. Through high-throughput screening of small molecule compounds, certain 1-benzylpiperidin-4-ol derivatives (Figure 9) have been identified as inhibitors of type I DHOases, such as those from *Bacillus anthracis* and *Staphylococcus aureus* [45,46]. These inhibitors may leverage the differences in loop length and residue composition among the three types of DHOases. For instance, the shorter loop in type I DHOases contains a glycine residue whose peptide backbone forms the necessary hydrogen bonds with the substrate, replacing the two threonine residues found in type II DHOases (Figure 10). However, due to significant differences in structural and biochemical properties across DHOase types, it remains largely unexplored whether or not inhibitors such as afatinib, 5-fluoroorotate, and natural products like plumbagin, kaempferol, and their derivatives can universally inhibit all DHOases. For example, while 5-fluoroorotate is a potent inhibitor of *Plasmodium falciparum* DHOase [41,43], its lack of efficacy against type I DHOases such as *Bacillus anthracis* DHOase remains unexplained at both structural and mechanistic levels [96]. Plumbagin exhibits a major inhibitory effect on type II DHOases from pathogens but has only a minor effect on human DHOase [73]. Thus, further investigation into the unique active site dynamics, regulatory mechanisms, and structural differences of DHOases across species is critical. Such research holds significant promise for developing innovative treatments for cancer, parasitic infections, and viral diseases by enabling the design of selective and species-specific inhibitors.

## 3. The Loop-In Binding Mechanism for Ligand Binding and Therapeutic Targeting

### 3.1. Revisiting the Loop-In Binding Mode

DHOase plays a crucial role in the de novo pyrimidine biosynthesis pathway by catalyzing the cyclization of *N*-carbamoyl-L-aspartate to dihydroorotate (Figure 1). The type II DHOase from *Escherichia coli* was the first to have its structure determined [57], providing invaluable insights into the reaction mechanism [67]. Crystallographic studies revealed that the substrate, *N*-carbamoyl-L-aspartate, and the product, dihydroorotate, occupy distinct sites within the enzyme dimer. Further structural investigations demonstrated that a flexible active site loop extends toward the substrate-binding pocket upon *N*-carbamoyl-L-aspartate binding (loop-in mode) and retracts during product release (loop-out mode) [57,58]. Non-substrate ligands, such as the product-like inhibitor 5-fluoroorotate, were also categorized under the loop-out mode based on structural evidence [58]. Additionally, mutagenesis studies have identified two critical threonine residues (T109 and T110) on this flexible loop, which are essential for catalytic function, as supported by structural and biochemical analyses [97]. These studies collectively suggested that the active site of DHOase operates via a dynamic loop mechanism, wherein the loop-in conformation stabilizes substrate binding and facilitates catalysis, while the loop-out conformation is associated with product release.

**Figure 11:**
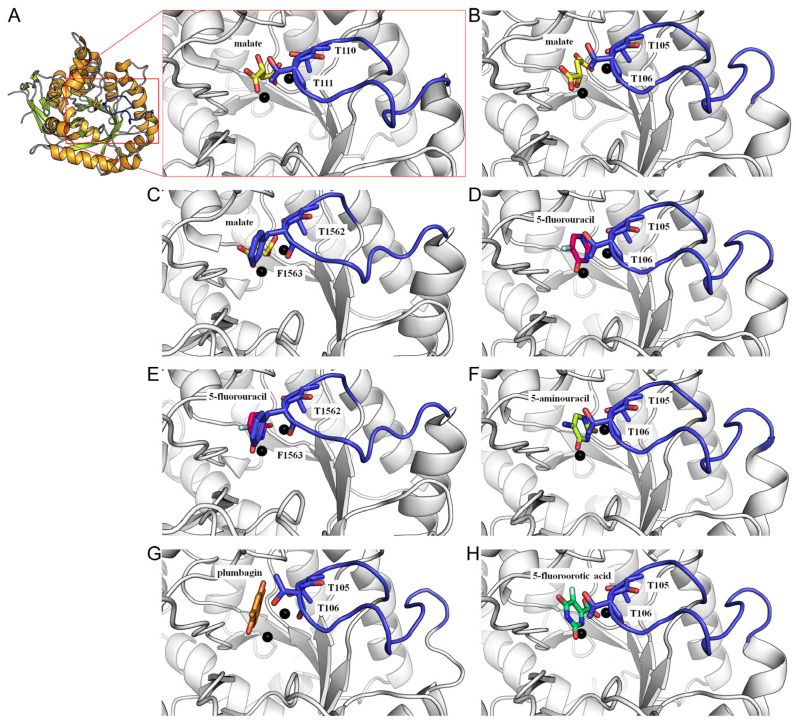
The loop-in binding mode. (**A**) The complex structure of *Yersinia pestis* DHOase bound to malate (PDB ID: 6CTY). The flexible loop residues 107–118 are highlighted in blue. Zinc ions in the active site are represented as black spheres. (**B**) The complex structure of *Saccharomyces cerevisiae* DHOase bound to malate (PDB ID: 7CA1). The flexible loop residues 102–116 are shown. (**C**) The complex structure of *Homo sapiens* DHOase domain bound to malate (PDB ID: 8GW0). The flexible loop residues 1560–1569 are shown. (**D**) The complex structure of *Saccharomyces cerevisiae* DHOase bound to 5-fluorouracil (PDB ID: 6L0B). (**E**) The complex structure of *Homo sapiens* DHOase domain bound to 5-fluorouracil (PDB ID: 8GVZ). (**F**) The complex structure of *Saccharomyces cerevisiae* DHOase bound to 5-aminouracil (PDB ID: 6L0F). (**G**) The complex structure of *Saccharomyces cerevisiae* DHOase bound to plumbagin (PDB ID: 7CA1). (**H**) The complex structure of *Saccharomyces cerevisiae* DHOase bound to 5-fluoroorotate (PDB ID: 7CA0).

However, multiple lines of evidence suggest that this model may be more complex than previously assumed, particularly given the structural and mechanistic differences among the three types of DHOases. First, the sequence composition and length of the flexible loop in type I *Bacillus anthracis* DHOase [46] and type III huDHOase [44] are significantly distinct from that of *Escherichia coli* DHOase, raising the question of whether the loop functions in a similar manner across all DHOases. Second, chimeric huDHOase containing the *Escherichia coli* DHOase flexible loop was found to be catalytically inactive, indicating species-specific catalytic requirements and mechanistic divergence [69]. Third, recent crystallographic evidence challenges the classical loop-in/loop-out model, revealing that certain non-substrate ligands and inhibitors interact with DHOase through a loop-in conformation rather than the previously assumed loop-out mode [48,68,71,72,73,74]. Notably, minor inhibitor malate (Figure 11A–C), the clinical anticancer drug 5-fluorouracil (Figure 11D,E), the thymine antagonist 5-aminouracil (Figure 11F), and potent inhibitors such as plumbagin (Figure 11G) and 5-fluoroorotate (Figure 11H) all exhibit loop-in binding.

Importantly, despite its structural similarity to dihydroorotate, 5-fluoroorotate binds *Escherichia coli* DHOase via the loop-out mode, where the active site loop does not engage with the ligand or the surrounding catalytic residues [58]. In contrast, *Saccharomyces cerevisiae* DHOase, despite also being a type II enzyme, binds 5-fluoroorotate through a distinct loop-in mechanism, where the ligand adopts a reverse orientation compared to its binding mode in *Escherichia coli* DHOase. This unexpected finding suggests that the traditional loop-in/loop-out model may not universally apply across all DHOases and necessitates a reassessment of the enzyme’s reaction mechanism, particularly in the context of drug development. These structural differences underscore the potential for designing species-specific inhibitors targeting distinct forms of DHOase. Given the essential role of DHOase in pyrimidine and UMP biosynthesis, the development of selective inhibitors tailored to specific pathogens and cancer cells holds significant promise for therapeutic applications.

### 3.2. Therapeutic Implications of the Loop-In Binding Mode

Recent findings suggest that the loop-in binding mechanism may be a conserved feature in certain eukaryotic DHOases, such as those from *Saccharomyces cerevisiae* [68,72,73,74] and humans [44,71], providing new insights into their catalytic cycles. The discovery of the loop-in mode as a primary ligand-binding mechanism in DHOase opens new avenues for drug development. Notably, in the loop-out mode, inhibitors do not interact with the flexible loop, potentially leading to weaker stabilization of the inhibition. In contrast, the loop-in mode provides additional interactions with inhibitors, enhancing binding affinity and efficacy. Targeting the active site loop offers a promising strategy for designing selective inhibitors that stabilize the loop in a closed conformation, thereby preventing substrate turnover. This approach is particularly relevant for diseases where pyrimidine biosynthesis is dysregulated, such as cancer and infectious diseases. In cancer therapy, the lung cancer drug afatinib has been reported to inhibit CAD activity [30], yet its precise mechanism of inhibition remains unclear and requires further structural characterization. Given recent structural insights, afatinib and related inhibitors could be optimized to specifically target the loop-in conformation, enhancing their efficacy in disrupting nucleotide biosynthesis in tumor cells. For antimicrobial applications, the observation that 5-fluoroorotate, a well-known antimalarial drug, strongly inhibits *Plasmodium falciparum* DHOase but lacks activity against *Bacillus anthracis* DHOase suggests that species-specific differences in the loop-in mechanism influence inhibitor binding [46]. To develop inhibitors that selectively target pathogens while minimizing off-target effects on huDHOase, it may be necessary to avoid interactions with the flexible loop in huDHOase, particularly with catalytically important residues such as F1563 (Figure 10). These structural differences present an opportunity to design antimicrobial agents that specifically inhibit pathogen DHOases while sparing host enzymes.

To translate these findings into therapeutic applications, further structural and biochemical investigations are essential to fully elucidate the conformational dynamics of the active site loop across different DHOases. Expanding the dataset of DHOase–inhibitor complexes will refine our understanding of ligand interactions and facilitate the rational design of novel inhibitors. Additionally, computational studies can model the conformational transitions between loop-in and loop-out states, identifying potential allosteric sites for drug targeting. Understanding the relationship between structure and inhibition will enable the structure-guided optimization of existing inhibitors to enhance interactions with loop-in residues, ultimately leading to the development of highly selective and potent DHOase inhibitors.

### 3.3. The Loop-In Binding Mode Observed in DHPase and ALLase

The loop-in binding mode is not exclusive to DHOase but is also observed in DHPase [56] and ALLase [85,88]. DHOase, DHPase, and ALLase belong to the cyclic amidohydrolase family, a group of metal-dependent enzymes that catalyze the hydrolysis of cyclic amide bonds in either five- or six-membered rings (Figure 5), playing essential roles in purine and pyrimidine metabolism [56,78]. In DHPase, a conserved tyrosine residue within a dynamic loop plays a critical role in stabilizing the tetrahedral transition state during substrate hydrolysis, facilitating the transition-state collapse, product formation, and product release [70,79,83,98,99]. This suggests that, similar to DHOase, the dynamic loop in these cyclic amidohydrolases is functionally significant and could serve as a promising drug target for inhibitor design.

Plumbagin has been recently identified as a strong inhibitor of both DHOase [73] and DHPase [80]. Although the crystal structure of the DHPase–plumbagin complex has not yet been determined, it is speculated that plumbagin may bind to the flexible loop, as in the case of DHOase, thereby inhibiting enzymatic activity of DHPase. Further structural analyses are necessary to elucidate the detailed architecture and functional mechanisms of different DHOases. This information will be critical for advancing drug development and optimizing inhibitors for therapeutic applications.

## 4. Conclusions

In recent studies, the conformational dynamics of the active site loop of dihydroorotase have emerged as a potential drug target. Structural analyses have revealed that the active site loop undergoes significant conformational changes that are essential for its catalytic activity. Targeting these conformational states with small molecules or inhibitors could disrupt the enzyme’s function, thereby impeding pyrimidine biosynthesis and cancer cell proliferation. This review aims to elucidate the role of the active site loop conformation in dihydroorotase as a drug target. The revised understanding of DHOase ligand binding through the loop-in mechanism challenges previous models and offers new directions for drug discovery. By exploiting the unique conformational dynamics of the active site loop, researchers can develop novel inhibitors targeting pyrimidine biosynthesis in cancer cells and microbial pathogens. These insights underscore the importance of structural biology in guiding therapeutic strategies and highlight the potential of DHOase as a promising drug target.
